# Identification and functional analysis of serine protease inhibitor gene family of *Eocanthecona furcellata* (Wolff)

**DOI:** 10.3389/fphys.2023.1248354

**Published:** 2023-09-18

**Authors:** Man Zhang, Zhenlin Dai, Xiao Chen, Deqiang Qin, Guoyuan Zhu, Tao Zhu, Gang Chen, Yishu Ding, Guoxing Wu, Xi Gao

**Affiliations:** ^1^ College of Plant Protection, Yunnan Agricultural University, Kunming, Yunnan, China; ^2^ Yunan Tobacco Company Chuxiong Prefecture Company, Chuxiong, China

**Keywords:** *Eocanthecona furcellata* (Wolff), natural enemy, salivary gland, serine protease inhibitor, RNA interference

## Abstract

The predatory natural enemy *Eocanthecona furcellata* plays a crucial role in agricultural ecosystems due to its effective pest control measures and defensive venom. Predator venom contains serine protease inhibitors (SPIs), which are the primary regulators of serine protease activity and play key roles in digestion, development, innate immunity, and other physiological regulatory processes. However, the regulation mechanism of SPIs in the salivary glands of predatory natural enemies is still unknown. In this study, we sequenced the transcriptome of *E. furcellata* salivary gland and identified 38 SPIs genes named *EfSPI1∼EfSPI38*. Through gene structure, multiple sequence alignment and phylogenetic tree analysis, real-time quantitative PCR (RT-PCR) expression profiles of different developmental stages and different tissues were analyzed. RNAi technology was used to explore the gene function of *EFSPI20*. The results showed that these 38 EfSPIs genes contained 8 SPI domains, which were serpin, TIL, Kunitz, Kazal, Antistasin, Pacifastin, WAP and A2M. The expression profile results showed that the expression of different types of EfSPIs genes was different at different developmental stages and different tissues. Most of the EfSPIs genes were highly expressed in the egg stage. The *EfSPI20, EfSPI21, EfSPI22*, and *EfSPI24* genes of the Pacifastin subfamily and the *EfSPI35* gene of the A2M subfamily were highly expressed in the nymphal and adult stages, which was consistent with the RT-qPCR verification results. These five genes are positively correlated with each other and have a synergistic effect on *E. furcellata*, and they were highly expressed in salivary glands. After interfering with the expression of the *EfSPI20* gene, the survival rate and predatory amount of male and female adults were significantly decreased. Taken together, we speculated some EfSPIs may inhibit trypsin, chymotrypsin, and elastase, and some EfSPIs may be involved in autoimmune responses. *EfSPI20* was essential for the predation and digestion of *E. furcellata*, and the functions of other EfSPIs were discussed. Our findings provide valuable insights into the diversity of EfSPIs in *E. furcellata* and the potential functions of regulating their predation, digestion and innate immunity, which may be of great significance for developing new pest control strategies.

## 1 Introduction

*Eocanthecona furcellata* is a natural enemy insect of Hemiptera, Pentatomidae. They can prey on Lepidoptera, Coleoptera and Hemiptera pests and have great application potential in biological control (Wen et al., 2017; Fan et al., 2019; Chen et al., 2021). It was found that the predation behavior of *E. furcellata* is similar to that of other predatory bugs such as *Arma chinensis* and *Betta macrostoma* (Wang et al., 2019; Wang et al., 2020). When searching for prey, they will quickly launch an attack, directly stabbing the mouth of the prey. At the same time, the saliva secreted by the salivary gland (also known as venom) is injected into the prey through the mouth needle to cause rapid paralysis of the prey (Lemos et al., 2005). In this process, they then use saliva to digest nutrients in their prey *in vitro* and absorb them back into the body through the needle (Cantón and Bonning, 2020). This digestion of predatory bugs allows them to prey on prey that is much larger than their size, expanding their predation range and biological efficiency, which is particularly important for the survival of predatory bugs (Cohen, 1990; Zhu, 2021).

The salivary gland is an insect organ that synthesises and secrete saliva, lubricating mouthparts and dissolves food. It was previously reported that the salivary gland structure of predatory bugs is conservative, mainly composed of the main and accessory glands. The main gland is divided into the anterior lobe of the main gland and the posterior lobe of the main gland. The main gland is connected to the mouth needle through the main gland duct, and the accessory gland is connected to the main gland through the accessory gland duct (Kumar and Sahayaraj, 2012). Similarly, the salivary glands of *E. furcellata* are composed of a smaller anterior lobe of the main gland, a larger elongated posterior lobe of the main gland, accessory glands between the anterior lobes of the main gland and the posterior lobes of the main gland, as well as hilum and U-folded accessory ducts (Suwanchainda and Kanost, 2009; Gao et al., 2020), the ultrastructure showed that their cell composition and structure were different, indicating that they secreted different salivary components and functions were also different (Gao et al., 2020). Many previous studies have shown that the saliva secreted by the anterior lobe of the main gland of predatory bugs mainly paralyses prey or defense. Whereas the saliva secreted by the posterior lobe of the main gland mainly plays a role in hydrolyzing prey tissues and digesting nutrients (Walker et al., 2017).

The saliva of predatory bugs has been identified as a source of toxins, including some digestive enzymes, proteins, peptides, etc. (Beak and Lee, 2014), which have the functions of paralyzing prey, defense, digesting nutrients, antibacterial, and lysing cells (Walker et al., 2016; Tonk et al., 2020; Wait et al., 2020). Currently, the research on the application of predatory bug venom mainly focuses on the Reduviidae bugs. Studies have found that predacious assassin bugs are injected with venom to capture prey or defend; for example, the venom of *Platymeris rhadamanthus* has a strong insecticidal effect, and injection into *Periplaneta americana* and *Drosophila melanogaster* can cause paralysis and death in a short time (Edwards, 1961; Walker et al., 2019). *Rhynocoris marginatus* venom can increase *S. litura* mortality and change its nutritional indicators, macromolecular number and digestive enzyme levels (Sahayaraj and Muthukumar, 2011). The venom of *Pristhesancus plagipennis* was injected into *Lucilia cuprina*, and paralysis and death could be produced after 15 min of application, while injection into crickets could immediately produce paralysis (Walker et al., 2018a). Four components isolated from the salivary glands of *Havinthus rufovarius* were injected into *L. cuprina*, resulting in paralysis and death (Wait et al., 2020). Previously, our laboratory found that the salivary gland secretion of *E. furcellata* had a lethal effect on the fourth instar larvae of *Spodoptera litura* and affected its detoxification enzyme activity. In general, except for toxicity studies of the salivary glands as a whole, only a peptide toxin was studied in the saliva of assassin bugs (Hemiptera: Reduviidae) (Corzo et al., 2001; Sahayaraj and Muthukumar, 2011). Therefore, it is necessary to further elucidate the venom protein components of *E. furcellata* and understand its venom function and insecticidal mechanism.

It has been reported that serine protease inhibitors (SPIs) are ubiquitous protein components in the saliva of predatory bugs (Beak and Lee, 2014; Walker et al., 2017; Walker et al., 2018a; Walker et al., 2019; Rügen et al., 2021), which is released into the prey body with the secretion of saliva during its predation process, and plays an important role in its defense and predation. SPIs are a class of protease inhibitor superfamily molecules with conserved amino acid sequences, spatial structure and highly differentiated functions (Silverman et al., 2001). They are widely found in animals, plants and microorganisms (Wang et al., 2014). They interact with serine proteases to regulate many physiological processes in organisms, such as blood coagulation, tissue growth, inflammatory response and other processes (Irving et al., 2002; Meyer-Hoffert et al., 2010). There are a large number of serine protease inhibitors, which can be divided into four categories according to sequence homology and mechanism of action: serpins, canonical inhibitors, non-canonical inhibitors and alpha-2-macroglobulins (A2Ms) (Yang et al., 2017). Serpins are the most widely distributed and abundant superfamily, which mainly inhibit the activity of serine proteases (SPs) or cysteine proteases (CPs) (Zeng, 2018). The Serpin domain usually has three *ß*-sheets and seven to nine α-helixes folded into a reactive central loop (RCL) (Gettins, 2002). According to sequence homology, active center position and disulfide bond structure, canonical inhibitors are divided into different subfamilies, such as TIL, Kazal, WAP, amfpi, Bowman-Birk, Antistasin, Pacifastin, Kunitz (Krowarsch et al., 2003; Zhao et al., 2012). Non-canonical inhibitors are only found in blood-sucking organisms, such as hirudin, tick anticoagulant peptide and ornithodorin, which have specific and strong inhibitory effects on thrombin and coagulation factors (Krowarsch et al., 2003). A2Ms are a class of broad-spectrum protease inhibitors, members of the thioester-containing proteins (TEPs) superfamily (Blandin and Levashina, 2004). They can inactivate various proteins, such as serine protease, cysteine protease, aspartic protease, papain and metalloproteinase (Thomas et al., 1989; Armstrong et al., 1999).

At present, SPIs genes of many insects have been identified and studied. A total of 80, 61 and 57 SPIs genes were identified in *Pteromalus puparum*, *Plutella xylostella*, and *Bombyx mori*, respectively (Zhao et al., 2012; Lin et al., 2017; Yang et al., 2017), 32 and 29 serpin family genes were identified in *Manduca sexta* and *D. melanogaster*, respectively (Hashimoto et al., 2003; Li et al., 2018). Most SPIs, as negative regulators of SPs, play an important role in inhibiting serine protease cascade activation pathways and maintaining homeostasis (Irving et al., 2000). For example, SPIs act as negative regulators in the melanization response of insect humoral immunity by inhibiting serine protease activity and negatively regulating the PPO activation pathway (An et al., 2013). In *M. sexta*, serpins1-serpins7 inhibited 14 serine protease genes in the PPO activation pathway (An et al., 2011). The PO activity in the hemolymph of *D. melanogaster* was significantly increased after deleting Spn27A, and the uncontrollable melanization and semi-lethal phenomenon occurred, indicating that the gene negatively regulated the PPO activation pathway (Ligoxygakis et al., 2002). Spn28D can effectively inhibit the melanization reaction induced by microorganisms, and the hemolymph will undergo a self-melanization reaction after deleting this gene (Scherfer et al., 2008). In parasitic wasps, the KAZAL family genes NvKSP-1, NvKSP-2 and a Pacifastin family gene NvPPI from *Nasonia vitripennis* inhibited PPO activation and trypsin activity (Qian et al., 2015; Qian et al., 2017).

SPIs can also negatively regulate the Toll signaling pathway, affecting the insect’s expression of antimicrobial peptides. The Serpin43Ac gene in *D. melanogaster* regulates the Toll pathway related to adult defense against pathogenic fungal infection (Levashina et al., 1999). Sometimes, the PPO activation pathway of some insects by SPIs overlaps with the Toll signaling pathway. SPN40, SPN48, SPN55 and SPN93 of *Tenebrio molitor* and Bmserpin-15 of *B. mori* can inhibit the activation of PPO in hemolymph and inhibit the production of antimicrobial peptides in hemolymph tissues (Jiang et al., 2009; Jiang et al., 2011; Liu et al., 2015). MmvSPN-1 and MmvSPN-2 from *Microplitis media* inhibited host PPO activation and antimicrobial peptide synthesis (Zhou et al., 2023), Lmserpin1 of *Locusta migratoria manilensis* is involved in regulating hemolymph melanization and promoting antimicrobial peptide synthesis (Li et al., 2021). SPIs with inhibitory activity can also play other functions. Serpin27A in *M. sexta* regulates ventral-dorsal axis formation (Hashimoto et al., 2003). Serpin4A of *Drosophila* is an intracellular protein that regulates protein processing in the cell secretory pathway (Richer et al., 2004). On the other hand, a small number of SPIs have no inhibitory activity, and they play a role similar to hormone precursor proteins, transporters, molecular chaperones and storage proteins (Irving et al., 2000; Zsila, 2010; Carrell et al., 2011).

Since SPIs play an important role in many biological processes, it is extremely important to identify SPIs family genes for a better understanding of their biochemical and molecular functions. In this study, based on the RNA-seq data of salivary glands of *E. furcellata*, the SPIs genes were identified, and their expression profiles were analyzed. To further study the function of SPIs genes in *E. furcellata*, we selected the *EfSPI20* gene with the highest expression level in the transcriptome for RNAi experiments. We analyzed the effect of the *EfSPI20* gene on *E. furcellata*, aiming to provide a theoretical basis for elucidating the functions of *E. furcellata* SPIs in digestion, innate immunity, development and reproduction.

## 2 Materials and methods

### 2.1 Insect rearing

*Eocanthecona furcellata* was collected from Yuanjiang County, Yunnan Province, China, under environmental conditions of temperature 27°C ± 1°C, relative humidity of 60%–80%, photoperiod of 14/10 h (light/dark), and fed on *T. molitor* pupae in the laboratory for multiple generations.

### 2.2 Sample collection and RNA extraction

The salivary glands of the 1–5 instar nymphs, male and female adults of *E. furcellata,* were dissected using anatomical tweezers and anatomical needles in PBS buffer solution under the Nissan Olympus binocular stereo anatomical mirror (Su et al., 2023). There were 3 replicates per sample, 80 salivary glands per replicate, and 50 eggs per replicate were collected. The salivary glands were transferred to a 1.5 mL RNase-free centrifuge tube containing 200 μL Trizol reagent. The salivary glands were fully ground into homogenate and added with Trizol to 1 mL for RNA extraction (RNAiso Plus Total RNA. Takara, Dalian, China). After RNA extraction, the purity of RNA was detected by agarose gel electrophoresis and NanoDrop2000 micro-ultraviolet spectrophotometer, respectively. After passing the test, it was sent to the Novogene Bioinformatics Technology Co., Ltd. (Beijing, China) for transcriptome library construction and sequencing.

### 2.3 Transcriptome sequencing, assembly, and gene annotation

A total of 1.5 μg qualified RNA samples were used for library construction. The size of the library was detected using an Agilent 2,100 bioanalyzer and sequenced on an Illumina Miseq Platform. The raw data obtained by sequencing were removed from the sequencing adaptor, ploy-N and low-quality sequences to obtain high-quality clean data (Bolger et al., 2014), and the contents of Q20, Q30 and GC were calculated by FastQC (version 0.11.9) software. High-quality sequencing data were obtained and assembled using Trinity (version 2.4.0) software. The assembled unigenes were compared in seven databases using BLAST software to obtain annotation information of functional genes (E-values < 1e^−5^).

### 2.4 Identification of serine protease inhibitor genes of *Eocanthecona furcellata*

We screened the homologous sequence of the serine protease inhibitor gene family from the salivary gland transcriptome of *E. furcellata* by the BLASTP program (E-value < 1e^−5^), and candidate gene sequences were obtained after deleting the redundant sequences. The ORFs (open reading frames) and conserved domain were predicted by NCBI (https://www.ncbi.nlm.nih.gov/), and the genes with complete serine protease inhibitor gene family domain were retained as the final identified serine protease inhibitor family genes in the transcriptome of *E. furcellata*.

### 2.5 Characterization and phylogenetic analysis

The online software Expasy (https://web.expasy.org/protparam/) was used to predict the iso-electric point and relative molecular weight of the amino acid sequence encoded by the SPIs genes. The online software SignalP5.0 (http://www.cbs.dtu.dk/services/SignalP/) was used to predict the SPIs amino acid sequence signal peptide. DNAMAN was used for multiple sequence alignment, and the phylogenetic tree was constructed using the adjacent ligation method in MEGA-X software (Kumar et al., 2016). The domains of SPIs were predicted by the NCBI CDD database (http://www.ncbi.nlm.nih.gov/Structure/cdd/docs/cdd_search.html) and visualized in TBtools (Chen et al., 2020).

### 2.6 Expression profiling and RT-qPCR analysis

#### 2.6.1 Detection of EfSPIs gene expression by RNA-seq

Clean reads were mapped to assembled transcriptomes using the default parameter Bowtie2 (version 2.2.6). The FPKM values (the number of transcript fragments per thousand bases per million reads) were obtained using TBtools (version 1.087) by sequencing the salivary gland transcriptomes (eggs, 1-5 instar nymphs, male and female adults) at different developmental stages and were used to draw the EfSPIs genes expression heat map with the average value of three biological replicates per sample. The Spearman correlation was used to calculate the correlation between genes and the FPKM levels of 38 genes extracted from the transcriptome data. Thirty-seven EfSPI genes with a threshold of Spearman’s coefficient >0.5 and *p* < 0.05 were screened. Based on the interaction relationship, the correlation regulatory network between different subtypes of gene families was constructed and visualized using Cytoscape (version 3.9.0) software.

#### 2.6.2 Detection of EfSPIs gene expression by RT-qPCR

We used RT-qPCR to verify the expression of candidate EfSPIs genes at different developmental stages (eggs, salivary gland of 1–5 instar nymphs, female adults, males) and different tissues (malpighian tubule, salivary gland, fat body, ovary, midgut, head, hemolymph). The total RNA from each tissue was extracted using a Trizol RNA extraction kit, and RNA concentration and quality were determined by spectrophotometer. The cDNA was synthesized with 1 μg of total RNA input using the Prime ScriptRT Reagent Kit with gDNA Eraser to remove gDNA (Takara, Dalian, China). Gene-specific primers were designed using the NCBI’s profile server, and the *EfRPL9* rRNA gene was used as a reference. RT-qPCR was carried out using the SYBR^®^ Premix EX Taq ™ II (Tli RNaseH Plus) (Takara, Dalian, China) on an Applied Biosystems 7,500/7,500 fast PCR system (ABI, Foster City, CA, United States). The reaction conditions were as follows: 95°C for 30 s, followed by 40 cycles of 95°C for 5 s, and 60°C for 30 s. Melting curve analysis was performed from 65°C to 95°C to determine the specificity of RT-qPCR primers. Each sample had three biological replicates. The 2^−ΔΔCT^ method (Schmittgen and Livak, 2008) was used to analyze gene expression profiles. All analyses were performed with three biological replicates. The primers used in RT-qPCR amplification are listed in Supplementary Table S1.

### 2.7 Effects of RNAi *EfSPI20* on the survival and feeding amount of *Eocanthecona furcellata*

To further study the function of serine protease inhibitors in *E. furcellata*, we selected the *EfSPI20* gene with highly expressed in the male and female adults and salivary glands of *E. furcellata* for RNA interference experiments Based on the cDNA sequences of *EfSPI20* and *GFP* genes, specific primers containing T7 promoter were designed using Primer 5.0 (Supplementary Table S1). The correct *EfSPI20* and *GFP* plasmid verified by cloning and sequencing were used as templates and PCR amplification was performed using GoldenStar^®^ T6 Super PCR Mix (1.1×) (Tsingke Biotechnology Co., Ltd. Beijing, China). The PCR products were detected by 1% gel electrophoresis, and the target fragments were recovered and purified by Trelief^®^ DNA Gel Extraction Kit (Tsingke Biotechnology Co., Ltd. Beijing, China). The purified DNA templates synthesised *dsEfSPI20* and *dsGFP* using an *in vitro* Transcription T7 Kit (Takara, Dalian, China). The integrity of dsRNA was evaluated by 1% agarose gel electrophoresis, and the concentration was measured by NanoDrop2000 micro-ultraviolet spectrophotometer. The dsRNA was diluted to 1 μg/μL with RNase Free H_2_O and stored at −80°C.

Male and female adults of *E. furcellata* within 3 days of feathering were selected for RNAi experiments. 1 μL dsRNA was slowly injected into the most lateral part of the intersegmental membrane of the posterior chest and abdomen of *E. furcellata* using a 1 μL micro-sampler. After injection, the test insects were transferred to a constant temperature incubator under controlled feeding conditions at 27°C ± 1°C temperature, relative humidity 60%–80%, and photoperiod 14/10 h (light/dark). The experimental group was injected with *dsEfSPI20*, and the control group was injected with *dsGFP*. Each group had 10 replicates and 5 test insects in each replicate, 5 pupae of *T. molitor* were fed, and the soaked absorbent cotton balls were used to provide. Salivary gland samples were collected at 1, 2, 4 and 6 days after injection to detect the expression level of *EfSPI20* in the salivary gland. As mentioned above, the *EfRPL9* rRNA gene was used as a reference for amplifying the RT-qPCR. Before injecting dsRNA into *E. furcellata*, the pupae of *T. molitor* were weighed and recorded. After 1 day of feeding, 5 pupae of *T. molitor* were weighed again, the daily food intake of each adult of *E. furcellata* was calculated, and the number of deaths was recorded. Then, the fresh pupae of *T. molitor* were weighed and placed in, and the next day were weighted, placed, and weighed again the next day. A total of 6 days of observation were recorded.

### 2.8 Statistical analysis and imaging

Graphs were made using GraphPad Prism (GraphPad Software, San Diego, CA, United States, version 8.0.2). Figures were designed using Adobe Illustrator 2021. Statistical significance was determined through one-way ANOVA, Tukey’s honestly significant difference (HSD) test or independent sample *t*-test, and *p*-values of *p* < 0.05 were considered significant.

## 3 Results

### 3.1 Transcriptome sequencing, assembly, and annotation

A total of 188,110,943 original sequences were sequenced from salivary gland samples of 1–5 instars nymphs and male and female adults, and 176,989,422 high-quality clean reads were obtained after 11,121,521 low-quality redundant sequences were removed. The total Clean Bases data size was 53.09G. The GC content of each sample ranged from 38.02% to 45.60%, with Q20 bases greater than 97.54% and Q30 bases greater than 92.94% (Supplementary Table S2). In total, 126,266 transcripts with an average length of 1,352 bp and N50 transcripts of 2,245 bp. Based on the transcript, 63,786 unigenes with a length of 68,027,300 bp and N50 with a length of 1,649 bp were obtained by further assembly. Among them, 18,837 unigenes with more than 1 kb were obtained, accounting for 29.53% (Supplementary Table S3).

### 3.2 Homology analysis and gene ontology annotation

**FIGURE 1:**
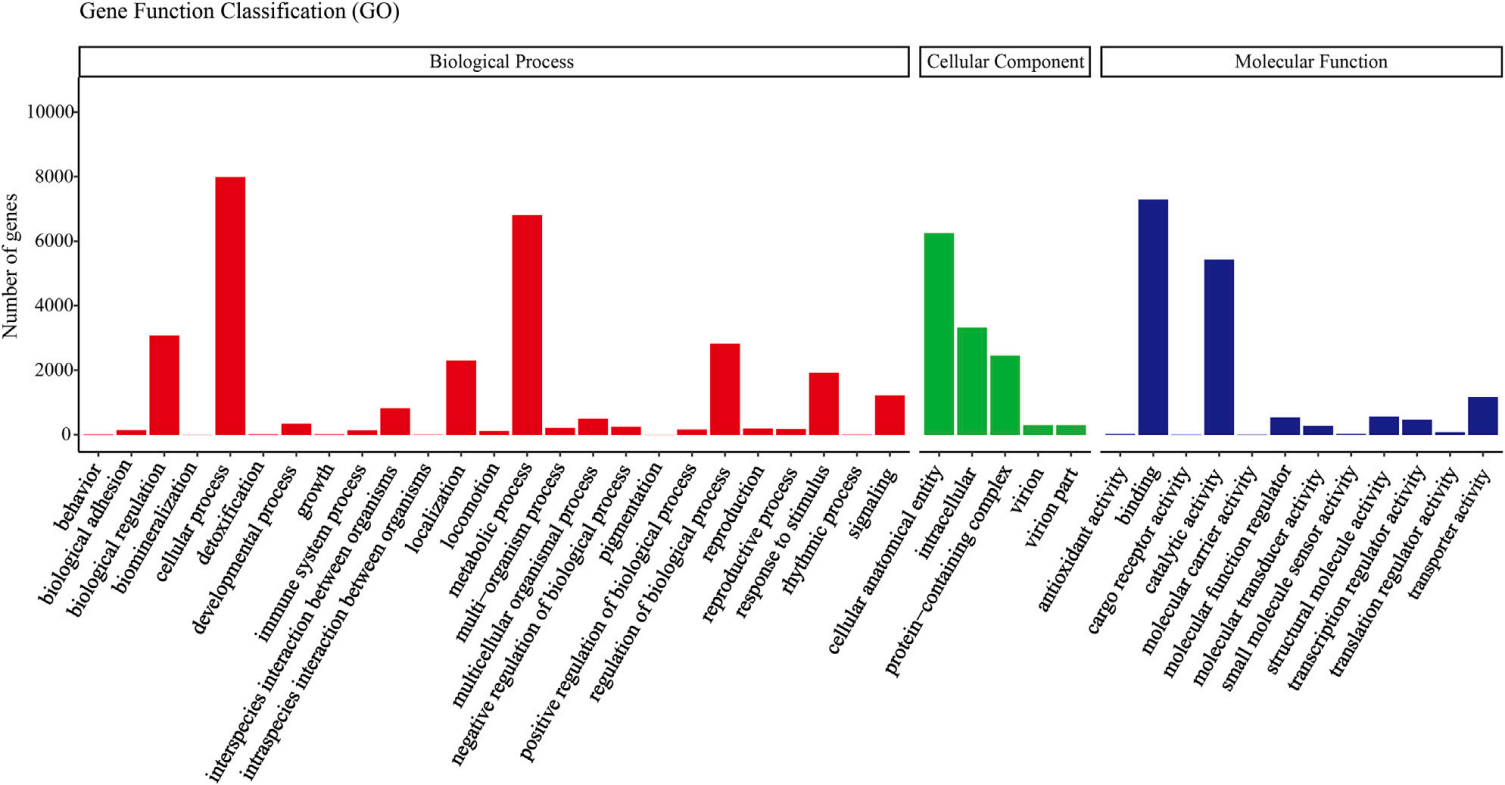
Gene ontology (GO) classification showed by the quantity of *Eocanthecona furcellata* transcripts.

All transcripts from *E. furcellata* were annotated in seven major databases (NR, Pfam, GO, NT, KO, KOG and Swiss-Prot). Among the 63786 transcripts, with a comment rate of 100%. 22,892 (35.88%) transcripts were annotated to the NR database, 14,391 (22.56%) to the Pfam database, 14,389 (22.55%) to the GO database, 12,576 (19.71%) to the NT database, 12,105 (18.97%) to the Swiss-Prot database, 7,447 (11.67%) to the KO database and 6,755 (10.59%) to the KOG database. NR database homology searches showed that the *E. furcellata* salivary gland transcriptome shared the similarity of unigenes with *Halyomorpha halys* was the highest (65%), followed by *Cryptotermes secundus* (4.34%). The transcripts annotated to the GO database were annotated to biological processes, cell components, and molecular functions. (Figure 1).

### 3.3 Identification and gene structure characteristics of EfSPIs gene family

Based on the reported insect SPI gene sequences, a total of 38 EfSPI genes were identified from the salivary gland RNA-seq of *E. furcellata* by homologous alignment, and they were named *EfSPI1∼EfSPI38*. The amino acid sequences encoded by these genes are 68–3,784 aa, and the theoretical molecular weight of the protein was quite different. The maximum was 416.99 kD, the minimum was only 7.35 kD, and the iso-electric point (PI) range was 4.08–10.26. Signal peptide prediction results showed that the amino acids of 19 EfSPIs genes had signal peptide sequences. These 38 EfSPIs genes contain eight domains, generally divided into three categories: serpin, canonical inhibitors and A2M (Table 1).

**TABLE 1:** Structural characteristics of EfSPIs.

Gene name	Domain	Category	Signal peptide	Amino acid/aa	Molecular mass/kD	Isoelectric point
EfSPI1	serpin	serpin	/	91	9.83	4.08
EfSPI2	serpin	serpin	1–20	407	45.58	7.37
EfSPI3	serpin	serpin	1–22	426	47.23	4.93
EfSPI4	serpin	serpin	/	1685	185.24	6.72
EfSPI5	serpin	serpin	1–31	856	94.28	8.86
EfSPI6	serpin	serpin	/	248	27.91	5.71
EfSPI7	serpin	serpin	/	396	44.58	9.83
EfSPI8	serpin	serpin	1–30	365	41.11	5.08
EfSPI9	serpin	serpin	1–20	416	46.72	8.71
EfSPI10	serpin	serpin	/	806	88.10	4.53
EfSPI11	serpin	serpin	/	331	38.51	9.06
EfSPI12	serpin	serpin	/	81	87.67	4.66
EfSPI13	TIL	canonical SPIs	/	3784	416.99	5.08
EfSPI14	Kunitz	canonical SPIs	1–43	866	96.91	6.43
EfSPI15	Kunitz	canonical SPIs	/	740	83.52	7.43
EfSPI16	Kunitz	canonical SPIs	/	1979	223.06	6.33
EfSPI17	Kunitz/WAP	canonical SPIs	/	2754	298.77	4.51
EfSPI18	WAP	canonical SPIs	1–22	410	46.35	10.02
EfSPI19	Antistasin	canonical SPIs	/	799	87.56	6.39
EfSPI20	Pacifastin	canonical SPIs	1–30	126	13.41	7.04
EfSPI21	Pacifastin	canonical SPIs	1–28	72	7.88	8.29
EfSPI22	Pacifastin	canonical SPIs	1–23	68	7.35	4.47
EfSPI23	Pacifastin	canonical SPIs	/	145	15.68	8.17
EfSPI24	Pacifastin	canonical SPIs	1–23	86	9.47	8.11
EfSPI25	KAZAL	canonical SPIs	1–26	234	25.32	4.73
EfSPI26	KAZAL	canonical SPIs	/	162	17.17	9.8
EfSPI27	KAZAL	canonical SPIs	1–31	293	34.10	4.87
EfSPI28	KAZAL	canonical SPIs	1–20	520	59.12	8.94
EfSPI29	KAZAL	canonical SPIs	1–27	505	58.08	5.88
EfSPI30	KAZAL	canonical SPIs	/	869	97.64	6.27
EfSPI31	KAZAL	canonical SPIs	1–26	942	104.81	6.74
EfSPI32	KAZAL	canonical SPIs	1–22	500	55.09	7.91
EfSPI33	KAZAL	canonical SPIs	/	133	14.76	10.26
EfSPI34	KAZAL	canonical SPIs	/	1613	173.78	7.02
EfSPI35	A2M	A2M	/	801	91.56	6.97
EfSPI36	A2M	A2M	1–22	759	88.05	7.12
EfSPI37	A2M	A2M	/	1668	191.05	6.76
EfSPI38	A2M	A2M	1–22	1539	171.20	6.35

### 3.4 Analysis of the amino acid domain and multiple alignments

**FIGURE 2:**
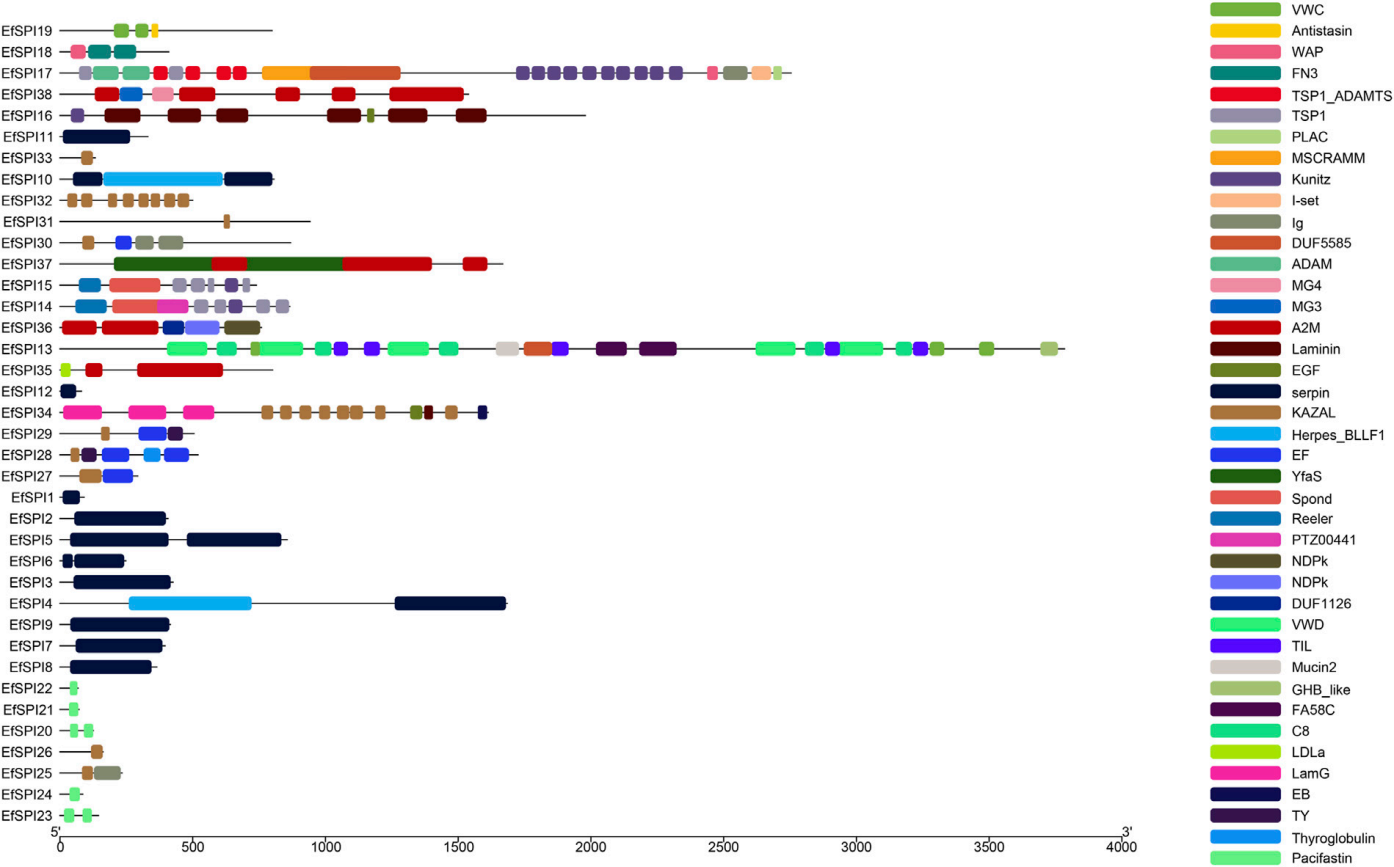
Protein conserved domain of SPIs genes of *Eocanthecona furcellata*.

Conserved domain analysis showed that 38 SPIs genes belong to serpin, TIL, Kunitz, Kazal, Antistasin, Pacifastin, WAP and A2M 8 subfamilies, and each subfamily contains one or more complete conserved domains. EfSPI1-3, EfSPI7-9, EfSPI11-12, EfSPI21-22, 24, EfSPI26, 31, 33 are single-domain proteins, and other EfSPIs are multi-domain proteins. EfSPI17 contains two SPIs family domains, including the Kunitz and WAP domains (Table 1, Figure 2).

**FIGURE 3:**
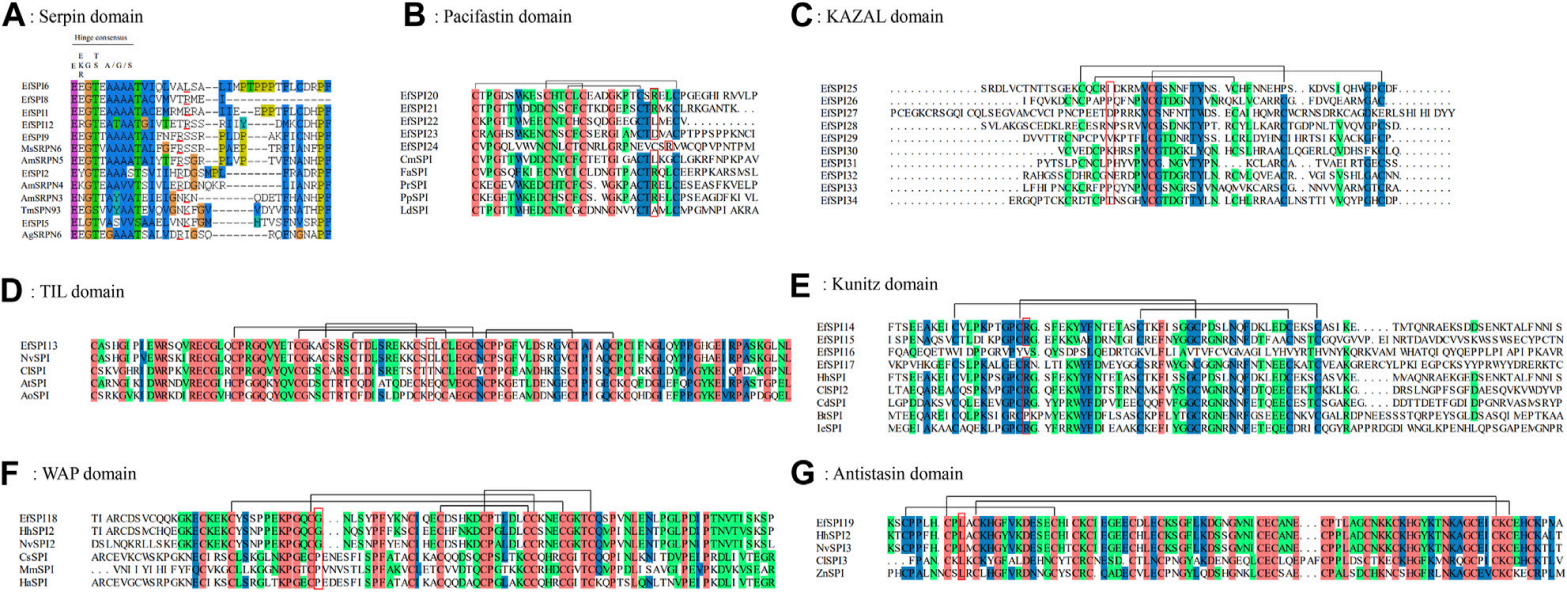
Multiple sequence alignment of the reactive center regions of the serpin subfamily and 6 subfamilies of canonical inhibitors of *Eocanthecona furcellata*. Predicted P1 residues are highlighted in red lines or red boxes. **(A)**: Serpin domain; **(B)**: Pacifastin domain; **(C)**: KAZAL domain; **(D)**: TIL domain; **(E)**: Kunitz domain; **(F)**: WAP domain; **(G)**: Antistasin domain. Species and Gene Bank accession numbers are shown in Supplementary Table S4.

To investigate the important structural regions of *E. furcellata* serpins, we aligned *E. furcellata* serpins with known inhibitory serpins from *Apis mellifera* (AmSRPN3, AmSRPN4, AmSRPN5), *Anopheles gambiae* (AgSRPN6), *M. sexta* (MsSRPN6) and *T. molitor* (TmSPN93). The result revealed that most residues in these structural regions (the breach, shutter and gate) in known inhibitory serpins were conserved in most *E. furcellata* serpins. The hinge regions of EfSPI3, EfSPI4, EfSPI7, EfSPI10 and EfSPI11 have no conserved amino acid residues, and the hinge regions of EfSPI1, EfSPI2, EfSPI5, EfSPI6, EfSPI8, EfSPI9 and EfSPI12 have conserved amino acid residues. The predicted P1 hydrolysis sites are Arg (R), Lys (K), or Leu (L) residues. EfSPI1, EfSPI6, EfSPI8, EfSPI9 and EfSPI12 were similar to the hinge region of MsSRPN6 and AmSRPN5, EfSPI2 was similar to the hinge region of AmSRPN4, and EfSPI5 was similar to the hinge region of TmSPN93 (Figure 3A, Supplementary Table S5).

In *E. furcellata*, canonical inhibitors accounted for the largest proportion, and 22 genes were identified, including six subfamilies: TIL, Kunitz, WAP, Antistasin, Pacifastin and KAZAL. The sequence alignment showed that the number and position of Cys residues in the same subfamily were highly conserved in the canonical inhibitors, and disulfide bonds were formed between them to maintain the structural stability of the protein. TIL has 5 pairs of disulfide bonds (Figure 3D). Pacifastin, KAZAL, Kunitz, and Antistasin have 3 pairs of disulfide bonds (Figures 3B, C, E, G). WAP has 4 pairs of disulfide bonds (Figure 3F). The amino acid residues at the hydrolysis site of P1 determine the specificity of the protein to the enzyme. The P1 sites of EfSPI20, EfSPI21 and EfSPI24 in the Pacifastin family were Arg (R), and the P1 site of EfSPI22 was Leu (L) (Figure 3B). The P1 sites of EfSPI14, EfSPI15 and EfSPI17 in the Kunitz subfamily were Arg (R) (Figure 3E). The P1 site of EfSPI18 in the WAP subfamily was Gly (G) (Figure 3F). The P1 site of EfSPI19 in the Antistasin subfamily was Leu (L) (Figure 3G).

### 3.5 Evolutionary analysis of EfSPIs

**FIGURE 4:**
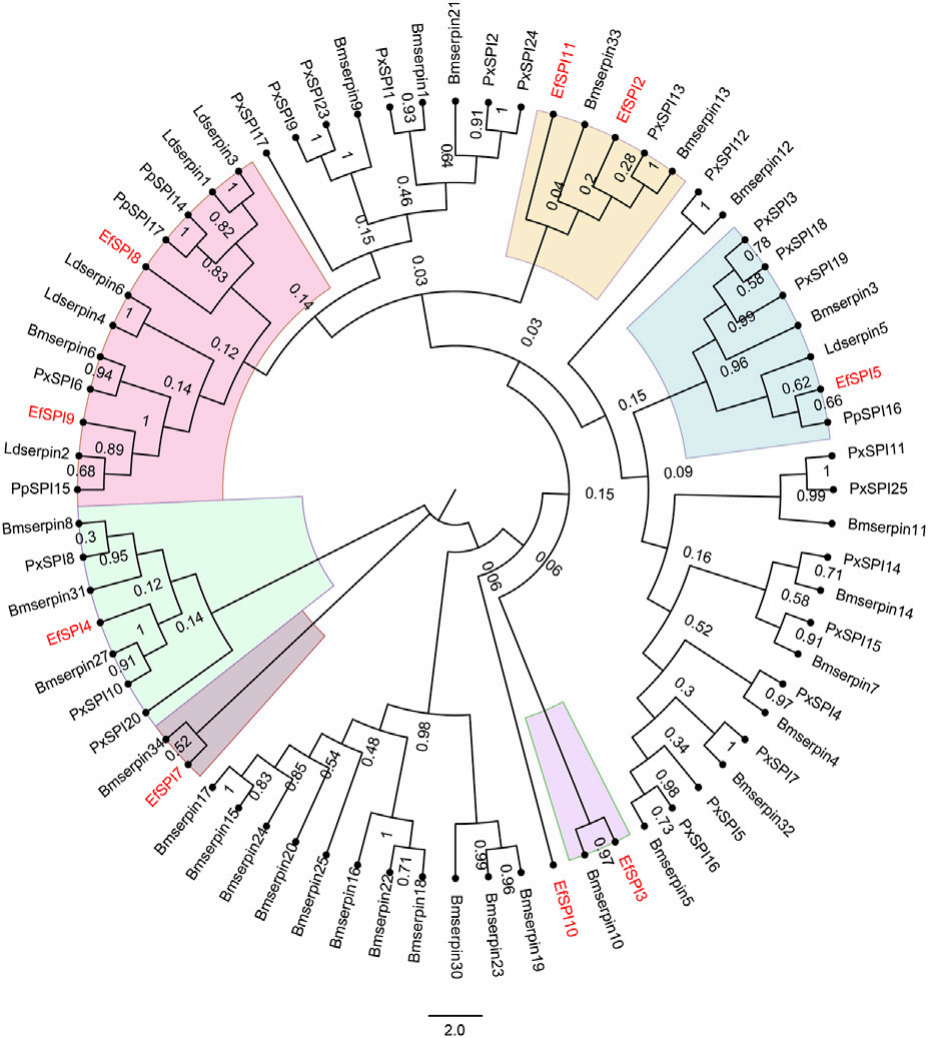
Phylogenetic relationship of serpins from *Eocanthecona furcellata* and four other insect species. The phylogenetic tree was constructed using the neighbor joining method for 1,000 times bootstrap replicates by MEGA 7.0.26 software. The first two letters in each of the serpins represent the acronym of the scientific name for a given species (Px: *P. xylostella*, Bm: *B. mori*, Ld: *L. distinctifemur*, Pp: *P. plagipennis*, Ef: *Eocanthecona furcellata*). The amino acid sequences used are shown in the Supplementary Material S1.

A phylogenetic tree was constructed with 83 serpins of *P. xylostella* (Lin et al., 2017), *B. mori* (Zhen et al., 2008), *Lethocerus distinctifemur* (Walker et al., 2018b), and *P. plagipennis* (Wait et al., 2020). It can be seen that the serpins of *E. furcellata* form seven branches. *EfSPI5*, *EfSPI8*, and *EfSPI9* have high homology with *L. distinctifemur* and *P. plagipennis*, indicating that the three serpins are highly conserved in Hemiptera. *EfSPI5* was clustered with *Ldserpin5* and *PpSPI16*. *EfSPI8* was clustered with *Ldserpin1*, *Ldserpin3*, *PpSPI14* and *PpSPI17*. *EfSPI9* was clustered with *Ldserpin2* and *PpSPI15*. *EfSPI2*, *EfSPI11* and *Bmserpin33*, *PxSPI13*, *Bmserpin13* clustered together. *EfSPI3* and *Bmserpin10* clustered together. *EfSPI7* and *Bmserpin34* clustered together. *EfSPI10* clustered alone (Figure 4, Supplementary Material S1).

**FIGURE 5:**
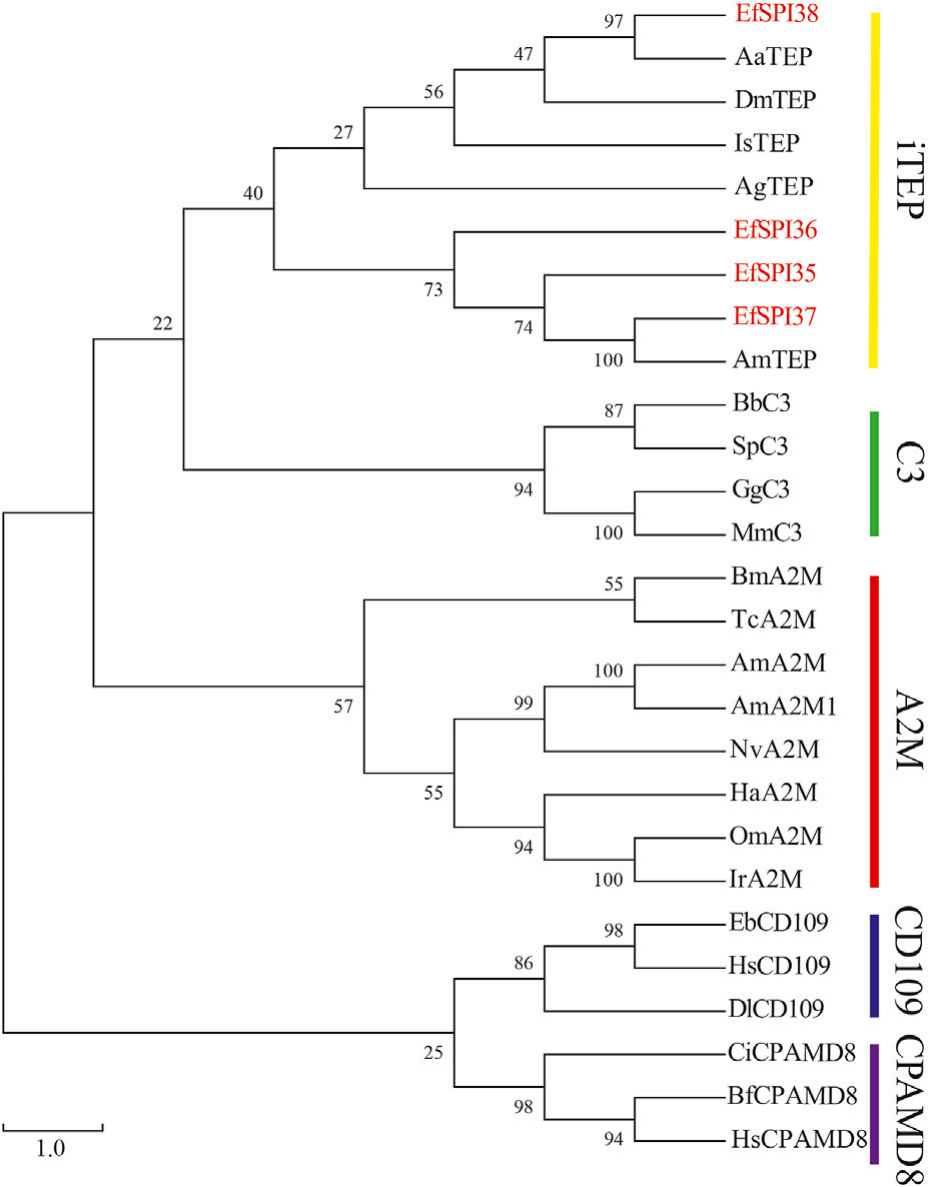
Phylogenetic analysis of thioester-containing proteins (TEPs) from *Eocanthecona furcellata* and other species. The phylogenetic tree was constructed using MEGA 7.0.26 with the neighbor joining method. The first two letters in each of the serpins represent the acronym of the scientific name for a given species. Species and Gene Bank accession numbers are shown in Supplementary Table S5.

Identified four EfSPIs genes of A2M subfamily, and by constructing phylogenetic trees with TEP family genes of different species known to classify, we can see that *EfSPI35*, *EfSPI36* and *EfSPI37* have high similarity with the corresponding protein sequence of *A. mellifera*, *EfSPI38* has high similarity with the corresponding protein sequence of *Aedes aegypti*, and *EfSPI35∼38* is clustered with iTEP gene (Figure 5, Supplementary Table S5).

### 3.6 Prediction of gene-gene interaction of EfSPIs

**FIGURE 6:**
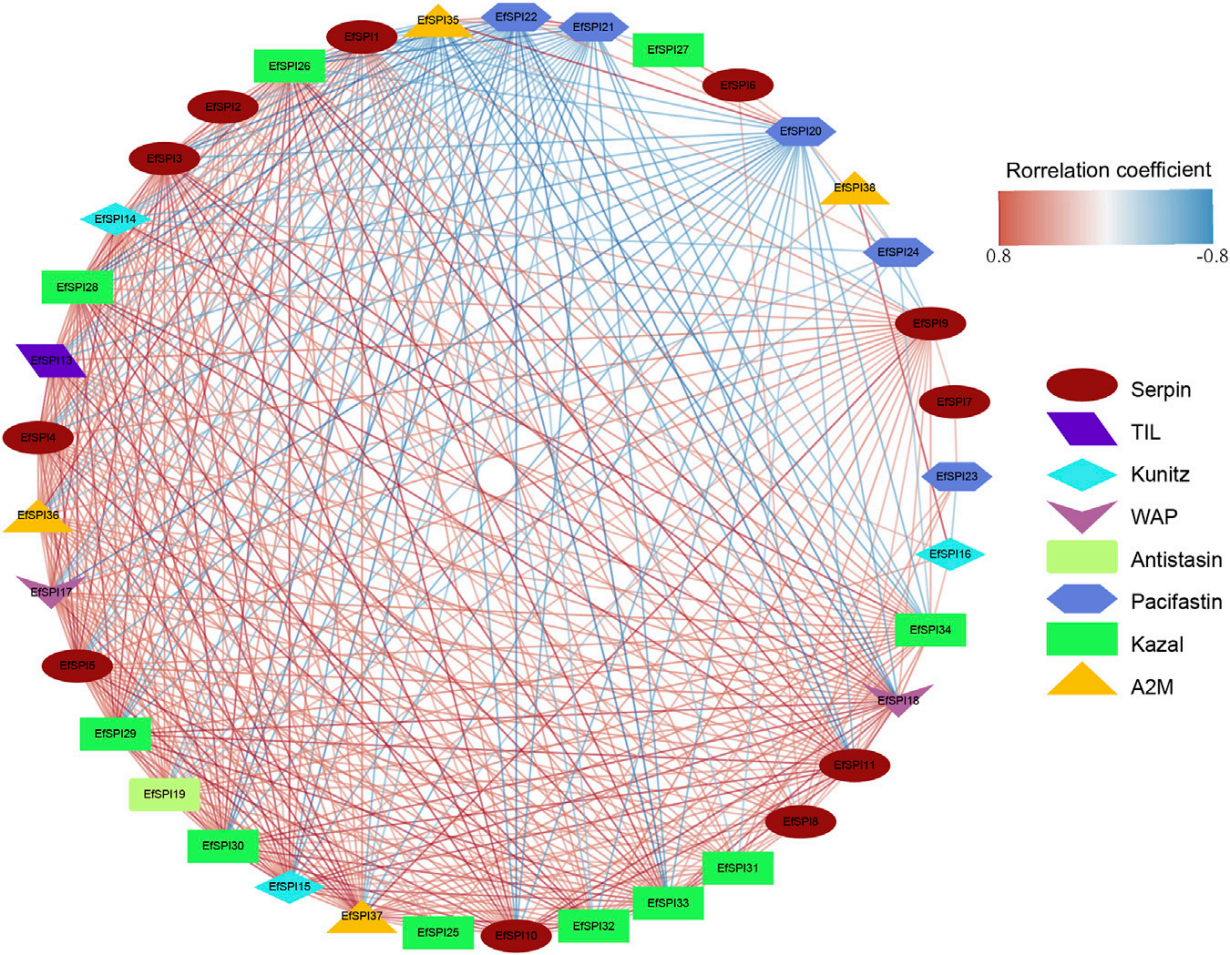
Correlation network of EfSPIs gene family members. The red line indicates a positive correlation, and the blue line indicates a negative correlation.

We constructed a gene-gene interaction network based on the FPKM expression of 38 *EfSPIs* genes to further study the interaction between different subfamily genes. As shown in Figure 6, the total network contains 37 nodes and 407 edges. It can be seen from the network diagram that there is a strong correlation between the members of the EfSPIs gene family, indicating that the members of the family interact closely, and most of the members are positively correlated. Only *EfSPI20*, *EfSPI21*, *EfSPI22*, and *EfSPI24* member genes in the Pacifastin family and *EfSPI35* gene in the A2M gene family were negatively correlated with most other family members, indicating that these five genes were regulated differently from other genes. To further screen the key genes in the network, the degree index of each gene in the network is calculated respectively. The larger the degree index, the more genes interact with the gene and the higher the importance in the network. The four most important ones are *EfSPI37*, *EfSPI5*, *EfSPI15* and *EfSPI3* (Supplementary Table S7).

### 3.7 Salivary gland of developmental and sex-specific expression

**FIGURE 7:**
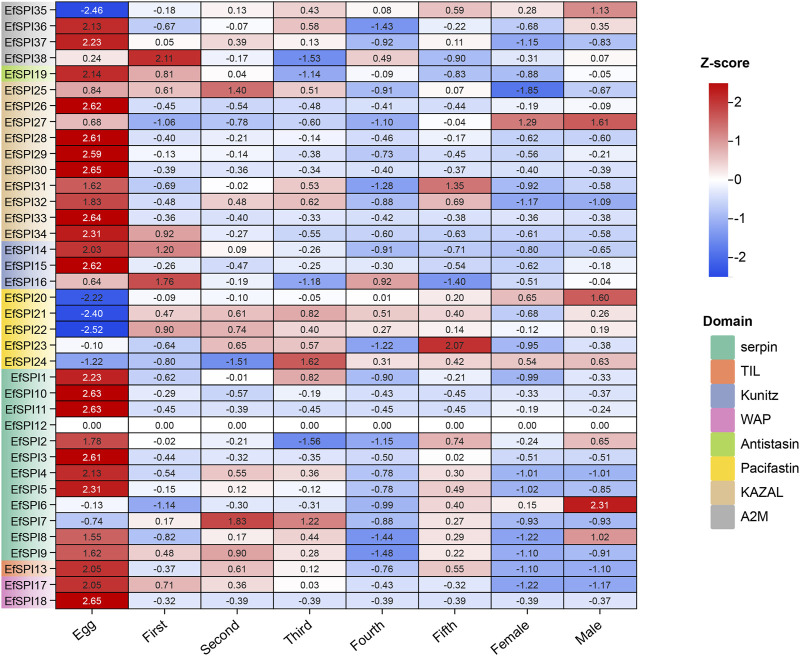
Expression profiles of EfSPIs genes across eggs and salivary glands of different developmental stages (1-5 instar nymphs, female and male adults). Egg: egg stage; First: first instar nymphs; Second: second instar nymphs; Third: third instar nymphs; Fourth: fourth instar nymphs; Fifth: fifth instar nymphs; Male: male adults; Female: female adults; each developmental stage had 3 replicates, the mean value of 3 repeated FPKM values was used to make the expression calorimetric map. The log2 (FPKM +1) value in Heat Map represents the gene expression level. The deeper red and blue indicated higher and lower expression levels, respectively.

We used RNA-seq data to analyze the *EfSPIs* gene family expression characteristics in the egg stage, salivary gland of 1–5 instar nymphs, and male and female adults. As shown in the figure, the expression of genes in different subfamilies was different in salivary glands of different developmental stages and male and female adults, and most genes are highly expressed in the egg stage. For example, *EfSPI36-37* (A2M), *EfSPI19* (Antistasin), *EfSPI25-34* (KAZAL), *EfSPI14-16* (Kunitz), *EfSPI1-5*, *EfSPI8-11* (serpin), *EfSPI13(TIL)*, *EfSPI17-18* (WAP) were highly expressed in egg stage. The *EfSPI20*, *EfSPI21*, *EfSPI22*, *EfSPI24* (Pacifastin) and *EfSPI35*(A2M) were only less expressed in the egg stage but highly expressed in the salivary gland of the nymph stage and adult stages. The *EfSPI12* (serpin) was almost undetectable throughout the salivary gland of the developmental period of *E. furcellata*, *EfSPI6* (serpin) was specifically expressed in the salivary gland of male adults, and *EfSPI7* (serpin) was specifically expressed in the salivary gland of the first and second instar nymphs. *EfSPI27* (KAZAL) was specifically expressed in the salivary glands of male and female adults. The EfSPI23 (Pacifastin) was specifically expressed in the salivary gland of the fifth instar nymphs. *EfSPI38* (A2M) was detected to be highly expressed in the salivary gland of the first instar nymphs (Figure 7).

**FIGURE 8:**
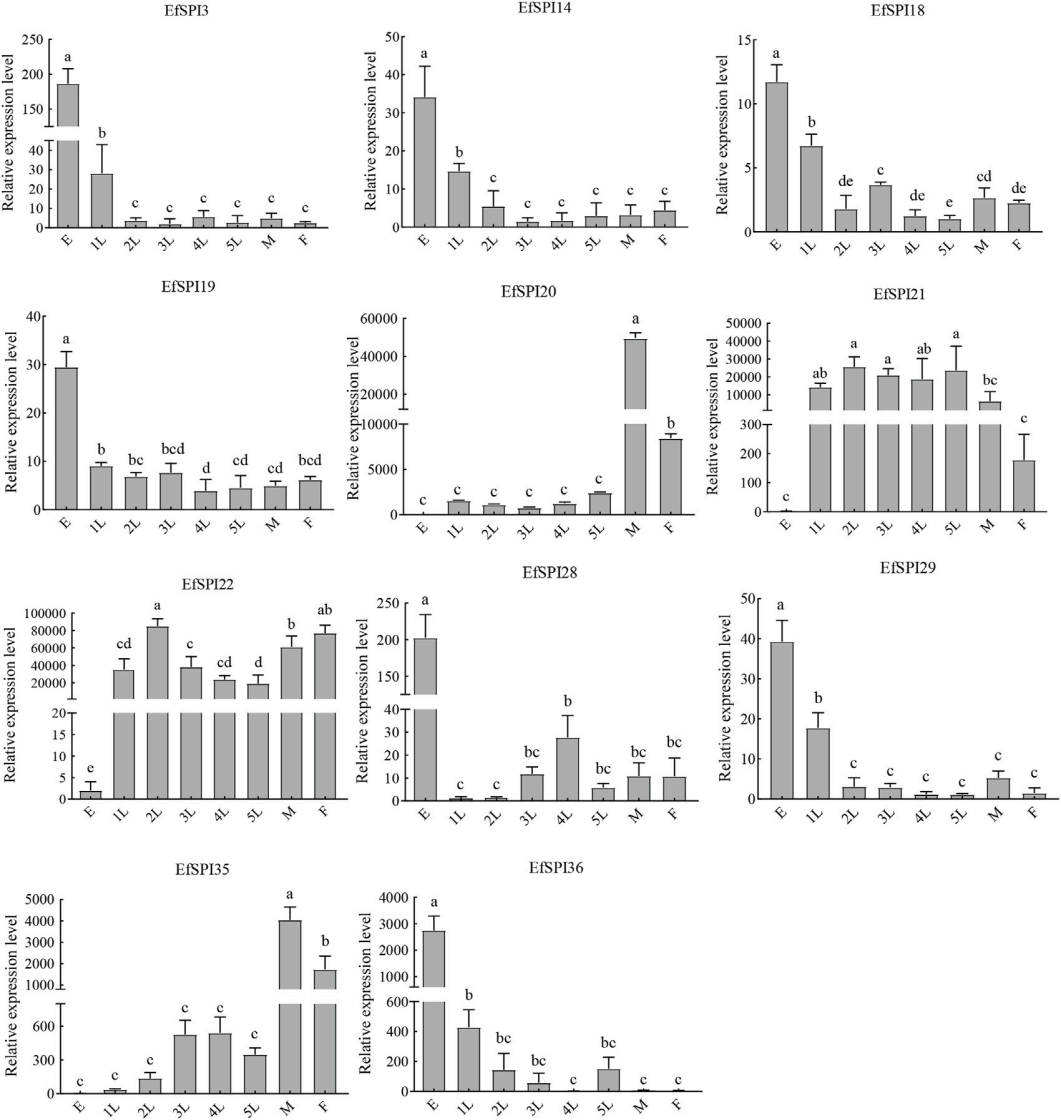
RT-qPCR of EfSPIs genes at different developmental stages. E: eggs, 1 L: first instar nymphs, 2 L: second instar nymphs, 3 L: third instar nymphs, 4 L: fourth instar nymphs, 5 L: fifth instar nymphs, M: male adults, F: female adults. *EfRPL9* rRNA was used as a housekeeping gene. Error bars represent the mean ± SE deviations from three biological replicates. A one-way ANOVA was used to determine the significant difference with different lowercase letters (a–e) (*p* < 0.05).

We selected *EfSPI3* (serpin), *EfSPI14* (Kunitz), *EfSPI18* (WAP), *EfSPI19* (Antistasin), *EfSPI20* (Pacifatin), *EfSPI21* (Pacifatin), *EfSPI22* (Pacifatin), *EfSPI28* (KAZAL), *EfSPI29* (KAZAL), *EfSPI35* (A2M), *EfSPI36* (A2M) from 38 EfSPIs genes to verify their expression levels in salivary glands. RT-qPCR was performed on eight developmental stages of eggs, 1-5 instar nymphs, and male and female adults. The results showed that the transcription of 11 EfSPIs genes was successfully detected in all developmental stages. *EfSPI3*, *EfSPI14*, *EfSPI18*, *EfSPI19*, *EfSPI28*, *EfSPI29* and *EfSPI36* were higher in the egg stage and lower in the nymph and adult stages. *EfSPI20*, *EfSPI21* and *EfSPI22* were highly expressed in nymphs and adults but almost not in eggs. The expression level of *EfSPI35* in male and female adults was significantly higher than in other developmental stages, and it was almost not expressed in the egg stage. Generally, the relative expression levels of most candidate EfSPIs at different developmental stages (Figure 8) were consistent with the FPKM values (Figure 7).

**FIGURE 9:**
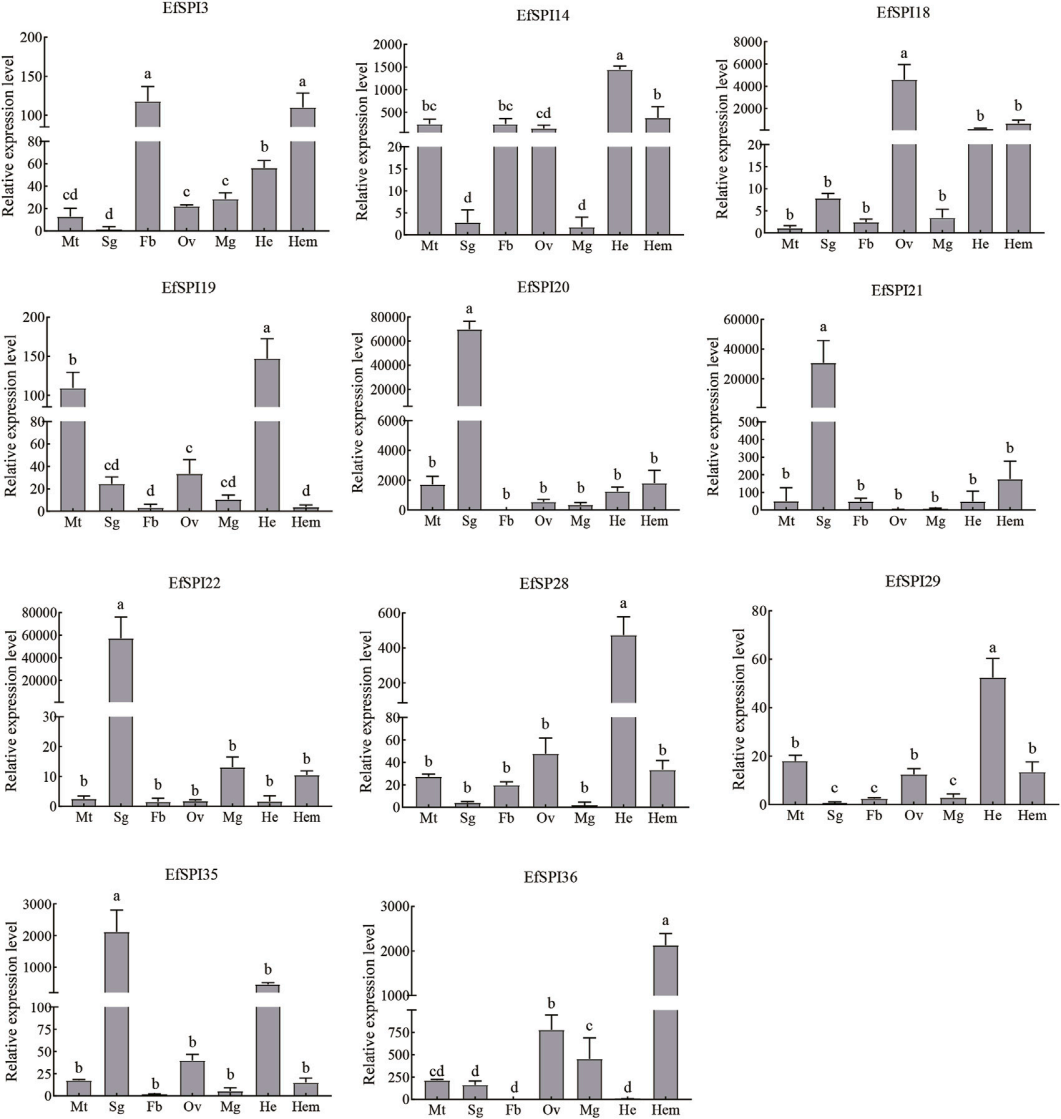
RT-qPCR of EfSPIs genes at different tissues. Mt: Malpighian tube, Sg: salivary gland, Fb: fat body, Ov: ovary, Mg: midgut, He: head, Hem: hemolymph. *EfRPL9* rRNA was used as a housekeeping gene. Error bars represent the mean ± SE deviations from three biological replicates. A one-way ANOVA was used to determine the significant difference with different lowercase letters (a–e) (*p* < 0.05).

Tissue expression results showed that transcription of 11 EfSPIs genes was detected in 7 tissues, including the Malpighian tube, salivary gland, fat body, ovary, midgut, head and hemolymph. *EfSPI3* was highly expressed in the fat body and hemolymph, followed by the head. *EfSPI4* was highly expressed in the head, hemolymph, malpighian tubule, fat body and ovary. *EfSPI18* was highly expressed in the ovary, followed by head and hemolymph. *EfSPI19* was highly expressed in the head and malpighian tubules. *EfSPI20*, *EfSPI21* and *EfSPI22* were highly expressed in salivary glands. *EfSPI28* and *EfSPI29* were highly expressed in the head, and *EfSPI35* was highly expressed in the salivary glands, followed by the head. *EfSPI36* was highly expressed in the hemolymph, followed by the ovary (Figure 9).

### 3.8 Detection of interference efficiency, survival rate and predation amount of *EfSPI20* gene in *Eocanthecona furcellata*

**FIGURE 10:**
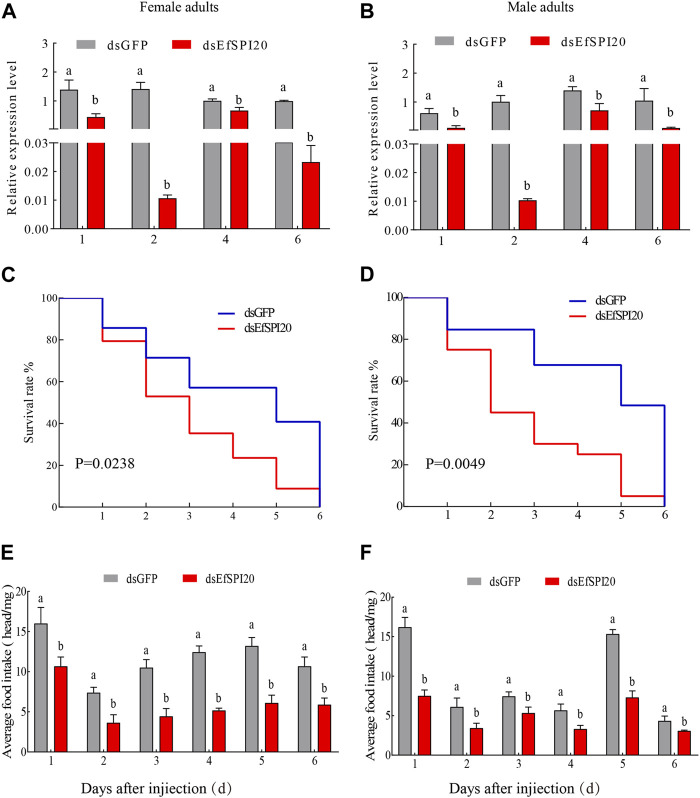
The transcription level, survival rate and predation amount of EfSPI20 in salivary glands of *Eocanthecona furcellata* after RNAi 1, 2, 4, 6 days. In the figure, **(A, B)** are the interference efficiency of female and male adults, respectively. **(C, D)** are the survival rates of male and female adults, respectively. **(E, F)** are the changes in the feeding amount of male and female adults, respectively. The data are expressed as mean ± SE; different letters indicate significant differences at the 0.05 level.

To further study the function of serine protease inhibitors in *E. furcellata*, we selected the *EfSPI20* gene highly expressed in the male and female adults and salivary glands of *E. furcellata* for RNA interference experiments. The interference efficiency of the *EfSPI20* gene in the salivary gland of *E. furcellata* is shown in Figure 10. Compared with *dsGFP* injection, the expression of the *EfSPI20* gene was significantly decreased after *dsEfSPI20* injection (*p* < 0.05). The expression levels of female adults (Figure 10A) decreased by 68.11%, 99.25%, 33.44% and 97.66%, respectively, after 1 day (*F* = 21.94, *p* < 0.05), 2 days (*F* = 112.14, *p* < 0.05), 4 days (*F* = 21.08, *p* < 0.05) and 6 days (*F* = 4263.20, *p* < 0.05) of injection. The expression levels of male adults (Figure 10B) decreased by 82.61%, 98.98%, 49.41% and 90.16%, respectively, after 1 day (*F* = 23.58, *p* < 0.05), 2 days (*F* = 65.99, *p* < 0.05), 4 days (*F* = 20.27, *p* < 0.05) and 6 days (*F* = 15.42, *p* < 0.05) of injection. Overall, the trend of interference efficiency of target genes in male and female adults was consistent, and the interference efficiency was the highest 2 days after injection.

The survival rate of *E. furcellata* after RNAi was counted and the results are shown in Figures 10C, D. Compared with the control, the survival rate of both sexes decreased significantly after interfering with the expression of the *EfSPI20* gene, and the survival rate decreased fastest on the second day after injection. The average survival time of male adults after injection of *dsGFP* was 4.12 ± 0.54 days, and the survival rate on the 4^th^ day was 57.10%. The average survival time of female adults after injection of *dsEfSPI20* was 3.00 ± 0.28 days, which was significantly lower than that of the control (*χ* ^*2*^ = 5.109, *p* < 0.05), and the survival rate on the 3^rd^ day was 40.70%. The average survival time of male adults after injection of *dsGFP* was 4.53 ± 0.55 days, and the survival rate on the 4^th^ day was 84.60%. The average survival time of female adults after injection of *dsEfSPI20* was 2.80 ± 0.37 days, which was significantly lower than that of the control (*χ* ^*2*^ = 7.928, *p* < 0.05), and the survival rate on the 2^nd^ day was 45.00%.

After RNAi expressing *EfSPI20*, the changes in feeding amount were detected. The results are shown in Figures 10E, F; compared with the control, the feeding amount of male and female adults after injection of *dsEfSPI20* was significantly lower than that of the control (*p < 0.05*). The average feeding amount of each female and male adult was 3.63 and 3.43 mg, respectively.

## 4 Discussion

The venom secreted by the salivary glands of predatory bugs can help them paralyze, hunt for prey and defend against natural enemies. However, the protein components in the saliva that can cause paralysis and death of prey are still unknown in *E. furcellata*. Therefore, this study determined and analyzed the transcriptome of the salivary glands based on the different stages of *E. furcellata* and identified the serine protease inhibitor family genes of the main components of the venom. A total of 38 EfSPIs genes were identified, belonging to 8 subfamilies: serpin, TIL, KAZAL, Kunitz, WAP, Antistasin, Pacifastin and A2M. Previous studies have shown that 57 PpSPI genes were identified in *P. puparum*, distributed in 7 subfamilies (Yang et al., 2017). 61 PxSPIs genes were identified in *P. xylostella* and distributed in 9 subfamilies (Lin et al., 2017). Eighty BmSPI genes were identified in *B. mori* and distributed in 10 subfamilies (Zhao et al., 2012), indicating that the SPIs protein type varies. The small number of SPIs gene families may be related to the fact that their family expansion only depends on tandem duplication (Wu et al., 2022). This might be due to the limitations of No-reference transcriptome analysis methods; some genes are not transcribed, or the low transcription level. There may be situations where they are not sequenced. In the future, we will use the whole genome sequencing assembly method to obtain the reference genome of the species to improve the transcriptome data.

Serpins are a structurally conserved and functionally diverse protein inhibitor superfamily (Irving et al., 2000). The protein sequence length, signal peptide, molecular weight and isoelectric point of the 12 EfSPIs genes in the serpin subfamily were different, consistent with the functional differentiation of the serpin gene family. Generally, the inhibitory serpin has a conserved RCL site, while the non-inhibitory RCL site is not. In the present study, the hinge regions of EfSPI1, EfSPI2, EfSPI5, EfSPI6, EfSPI8, EfSPI9 and EfSPI12 have conserved amino acid residues, so we predict that these genes may be inhibitory serpin. Studies have shown that the type of amino acid at the P1 site determines the inhibition specificity of serpin members. There are Arg (R) or Lys (K) residues at the P1 site, serpin acts on trypsin; when there are Phe (F), Tyr (Y), Leu (L) or Ile (I) residues at the P1 site, serpin acts on chymotrypsin; when there are Ala (A) or Val (V) residues at the P1 site, serpin acts on elastase (Sun et al., 2001). Through multiple sequence alignment, we found that EfSPI1, EfSPI2, EfSPI5, EfSPI8, EfSPI9, and EfSPI12 have an R or K residue at the P1 position, indicating that they may inhibit trypsin. At the same time, EfSPI6 contains an L residue at the P1 position, which may inhibit chymotrypsin. However, the hinge regions of EfSPI3, EfSPI4, EfSPI7, EfSPI10 and EfSPI11 have no conserved amino acid residues, so it is speculated that these genes may be non-inhibitory serpins and play a role in organisms such as molecular chaperones and hormone transport (Lin et al., 2017). Phylogenetic analysis further confirmed that the predicted five non-inhibited serpins were clustered with the predicted non-inhibited serpins of *P. xylostella* (Lin et al., 2017).

In addition, our study also found that EfSPI2, EfSPI11 and Bmserpin13 were clustered together, EfSPI4 and Bmserpin27 were clustered together, and EfSPI7 and Bmserpin34 were clustered together. The results of Zhen et al. (2008) showed that the mRNA levels of Bmserpin13, 27 and 34 in the fat body and blood cells increased after injection of bacteria into silkworm larvae, indicating that serine protease inhibitors play a regulatory role in defense response. It shows that EfSPI2, 4, 7, and 11 may play a similar role with these serpins of silkworms. EfSPI5, EfSPI8 and EfSPI9 are clustered with *L. distinctifemur* and *P. plagipennis* serpin*s*. Walker et al. (2018a; Walker et al., 2018b) showed that serpins of *L. distinctifemur* and *P. plagipennis* were isolated from venom and guessed that the venom protein paralyzed and killed the prey during the predation of *L. distinctifemur* and *P. plagipennis*, which contributed to their predation and digestion. As predatory natural enemies of Hemiptera, we speculate that EfSPI5, EfSPI8 and EfSPI9 of *E. furcellata* also have similar functions with serpins of *L. distinctifemur* and *P. plagipennis*; this remains to be verified in the next step.

In addition to the serpin subfamily, in this study, we identified 22 EfSPI genes of canonical inhibitors, which contain abundant Cys residues and form disulfide bonds to maintain the structural stability of proteins. The stable structures are necessary for keeping the right conformation to interact with the active site of serine proteases (Simonet et al., 2003). Like the serpin subfamily, the amino acid of the canonical inhibitors SPIs hydrolysis site P1 determines the inhibition of proteases. Unlike serpin, the process of canonical inhibitor proteins is reversible (Silverman et al., 2001). When the P1 site is Arg or Lys, it can inhibit trypsin enzyme activity. When it is Ala, Val or Gly, it can inhibit elastase enzyme activity. When it is Phe, Tyr, Leu or Met, it can inhibit chymotrypsin enzyme activity (Lin et al., 2017). The results showed that EfSPI14, EfSPI15, and EfSPI17 of the Kunitz subfamily may inhibit trypsin activity. The EfSPI18 of the WAP subfamily may inhibit elastase activity. EfSPI19 of the Antistasin subfamily may inhibit the activity of chymotrypsin. EfSPI20, EfSPI21 and EfSPI24 of the Pacifastin subfamily may inhibit trypsin activity, and EfSPI22 may inhibit chymotrypsin activity. However, some SPIs do not have inhibitory activity with the above inhibitory characteristics. For instance, the P1 site of NVPP-1 in *N. vitripennis* contains Arg residues but cannot inhibit trypsin activity, and the P1 site of NVPP-2 contains Met residues but cannot inhibit chymotrypsin activity (Qian et al., 2013). Therefore, whether the 9 EfSPIs predicted to inhibit trypsin, elastase or chymotrypsin in this study have the corresponding inhibitory activity needs further verified.

Gene expression profiles can provide important clues for gene function annotation. FPKM expression profile analysis showed that 25 EfSPI genes were highly expressed in the egg stage, such as EfSPI14, EfSPI18, EfSPI19, EfSPI28, EfSPI29, and EfSPI36, which were consistent with the results of RT-qPCR at the salivary gland of developmental stage. At the same time, they were also highly expressed or moderately expressed in the ovary, indicating that these EfSPI genes may be related to reproductive behaviors such as follicular development and ovulation (Pak et al., 2004). The five genes of EfSPI20, EfSPI21, EfSPI22, EfSPI24 (Pacifastin) and EfSPI35 (A2M) were almost not expressed in the egg stage but highly expressed in the salivary and of the nymph stages and female and male adults, and highly expressed in the salivary gland, thousands to 10,000 times higher than other tissues. It is reported that the predation behavior of *E. furcellata* mainly occurs in the nymph and adult stages. The first instar nymphs do not prey and can only complete their development by sucking plant juice. After entering the second instar, they begin to prey, and there is also the phenomenon of sucking plant juice. With the increase of instar, their phytophagous gradually weaken and their predatory gradually increases (Yao et al., 2019; Zhang et al., 2022). When preying on prey, *E. furcellata* secretes macromolecular substances such as salivary proteins produced by salivary glands into prey through oral needles to paralyze or kill prey and uses the secreted venom proteins to digest the nutrients in prey *in vitro* and then suck them back to the body for digestion by oral needles (Zhu, 2021; Su et al., 2023). The combined gene interaction network showed that the five genes were positively correlated, indicating they played a synergistic role in *E. furcellata*. Based on the above discussion, we speculate that these 5 genes may be closely related to the predation and digestion of *E. furcellata.*

Our study found that inhibition of *EfSPI20* expression significantly decreased the survival rate and food intake of *E. furcellata*. Combined with the results of previous studies, *EfSPI20* was highly expressed in the salivary gland, indicating that the salivary protein gene was essential for the growth, predation and digestion of *E. furcellata*. After the expression of the *Lmserpin1* gene was inhibited, the mortality of *L. migratoria manilensis* was significantly higher than the control (Li et al., 2021), consistent with this study’s results. As mentioned above, EfSPI20 may have the function of inhibiting trypsin. Trypsin is an important proteolytic enzyme in the digestive system of predatory insects (Agusti and Cohen, 2000). Their excessive hydrolysis or inhibited hydrolysis can cause digestive system disorders. Therefore, when the expression of EfSPI20 was interfered with, the digestive system of *E. furcellata* was disordered due to excessive hydrolysis or inhibited trypsin hydrolysis, resulting in a decrease in its survival rate and food intake. EfSPI20 is a member of the Pacifastin subfamily. It has been reported that the Pacifastin domain peptide in the venom of *N. vitripennis* can inhibit prophenoloxidase activation (Qian et al., 2013). The study of Walker et al. (2019) speculated that the pacifastin peptide of *P. rhadamanthus* may have similar activity to the Pacifastin peptide in the parasitoid venom and promote its feeding by preventing the hemolymph condensation of the prey. Therefore, it is not excluded that the Pacifastin gene in *E. furcellata* has such a function. After interfering with the expression of EfSPI20, it may affect the immune response and other physiological functions of *E. furcellata*, resulting in a decrease in its survival and predation. As a secreted venom serine protease inhibitor, whether EfSPI20 affects the growth and development, paralysis, and immunity of prey will be the next exploration goal of this study.

## 5 Conclusion

In general, this study provides some biological information about the serine protease inhibitors of *E. furcellata*, including gene structure, conserved domains, evolutionary relationships, and putative reaction sites. The expression profiles and RT-qPCR were used to understand the expression characteristics of EfSPIs at the salivary gland of different developmental stages, and the gene function of *EfSPI20* was preliminarily studied by RNAi technology. These results indicated that *EfSPI20* was essential for the predation and digestion of *E. furcellata*. These messages are crucial for the reproduction, development, reproduction, predation, digestion, immune response and application of *E. furcellata*. In the future, the corresponding protein products of EfSPI genes can be expressed and further explored for the above possible functions supporting the development of EfSPI protein products with insecticidal activity.

## Data Availability

The data presented in the study are deposited in the repository: https://www.ncbi.nlm.nih.gov/bioproject/PRJNA991139, accession number: PRJNA991139.
