# Supplementary material for: Identification and functional analysis of serine protease inhibitor gene family of Eocanthecona furcellata (Wolff)
Source: Front Physiol. 2023 Sep 18;14:1248354. doi: 10.3389/fphys.2023.1248354 (PMC10545863; doi:10.3389/fphys.2023.1248354)
Supplement: Supplementary file 1 [file DataSheet1.docx]

**Identification and functional analysis of serine protease inhibitor gene family of *Eocanthecona furcellata* (Wolff)**

**Man Zhang^1^, Zhenlin Dai^1^, Xiao Chen^1^, Deqiang Qin^1^, Guoyuan Zhu^1^, Tao Zhu^1^, Gang Chen^2^, Yishu Ding^2^, Guoxing Wu^1^, Xi Gao^1,*^**

^1^ Yunnan Agricultural University, Kunming 650201, Yunnan, China

^2^Yunan Tobacco Company Chuxiong Prefecture Company, Chuxiong 6750000, China

*** Correspondence:**Xi Gao
[chonchon@163.com](mailto:chonchon@163.com)

| **Table S1.** Amino acids for multiple sequence alignment with Serpin hinge region and active center loop region of *E. furcellata* | | |
| --- | --- | --- |
| Genes | Purpose | primer sequences (5'-3') |
| EfSPI3 | Real-Time PCR | CATTACGGTTGAGGACGGCT |
|  |  | AACAGCTTTTGCTCCCTCCA |
| EfSPI14 | Real-Time PCR | TTCGCATGCACAACACTTGG |
|  |  | CGTGTGGTATCTGGAGGACG |
| EfSPI18 | Real-Time PCR | GTGCTGATCAGATCGTGGCA |
|  |  | TGAGGCAAAGCCACCAGAAA |
| EfSPI19 | Real-Time PCR | TGACCAATCTGTTCCGAGCC |
|  |  | CGAGACACGTTCCTCCACAA |
| EfSPI20 | Real-Time PCR | AAGCAGTGGAGGGTCAATCG |
|  |  | AGGCTTACCGTCAGCTTCAC |
| EfSPI21 | Real-Time PCR | TTGCCGCAGAGGAAAGATGA |
|  |  | GCACTTCATGCGGGTACAAC |
| EfSPI22 | Real-Time PCR | GGTGAAGCTGCCTCTGTGAA |
|  |  | CATCCAAGTCGTTCCAGGCT |
| EfSPI28 | Real-Time PCR | CACAACAGTGAGGTTGGGGT |
|  |  | TTCTCGCAAAGGGTAGCTGT |
| EfSPI29 | Real-Time PCR | GAAGTATCGTTAGAACCCTCGT |
|  |  | ACAAATGAGCCAGGTTTTAGGAG |
| EfSPI35 | Real-Time PCR | AGTTTGGGCACTTGAGGCTT |
|  |  | CAGCTCCTTTGCCTACCACA |
| EfSPI36 | Real-Time PCR | CTGACATCGCACTGGCTCG |
|  |  | TTGTGGCTGTCAACTGTCGT |
| EfRPL9 | Real-Time PCR | TTCGAACCGCAGCAAGTTCT |
|  |  | GACGTTCAGGCATTTGGCAT |
| EfSPI20 | PCR amplification | AAGGTAGTGCTTGCTATTGC |
|  |  | ACCAGGTGTCTGTCGTGTAT |
| GFP | PCR amplification | GAGTGGTCCCAGTTCTTGTT |
|  |  | TGTCTTGTAGTTCCCGTCAT |
| dsEfSPI20 | RNAi | TAATACGACTCACTATAGGGAAGGTAGTGCTTGCTATTGC |
|  |  | TAATACGACTCACTATAGGGACCAGGTGTCTGTCGTGTAT |
| dsGFP | RNAi | TAATACGACTCACTATAGGGAGTGGTCCCAGTTCTTGTT |
|  |  | TAATACGACTCACTATAGGGTGTCTTGTAGTTCCCGTCAT |

| **Table S2.** Output statistics of *E. furcellata* transcriptome sequencing. | | | | | | |
| --- | --- | --- | --- | --- | --- | --- |
| Sample | Raw reads | Clean reads | Clean bases | Q20 percentage（%） | Q30 percentage（%） | GC Percentage（%） |
|  |  |  |  |  |  |  |
| First instar nymph | 22602826 | 21141236 | 6.34G | 98.1 | 93.95 | 44.43 |
|  |  |  |  |  |  |  |
| Second instar nymph | 23245159 | 22034569 | 6.61G | 98.19 | 94.11 | 44.39 |
|  |  |  |  |  |  |  |
| Third instar nymph | 25365926 | 24274453 | 7.28G | 97.54 | 92.94 | 43.65 |
|  |  |  |  |  |  |  |
| Fourth instar nymph | 23173151 | 21634340 | 6.49G | 98.09 | 93.99 | 45.43 |
|  |  |  |  |  |  |  |
| Fifth instar nymph | 23374405 | 21734234 | 6.52G | 98.31 | 94.45 | 45.51 |
|  |  |  |  |  |  |  |
| Female adult | 23650745 | 22242642 | 6.67G | 98.11 | 93.93 | 45.6 |
|  |  |  |  |  |  |  |
| Male adult | 23910217 | 22240040 | 6.67G | 98.22 | 94.16 | 44.86 |
|  |  |  |  |  |  |  |
| Egg | 22788514 | 21687908 | 6.51G | 97.79 | 93.03 | 38.02 |
|  |  |  |  |  |  |  |

| **Table S3.** Quality for de novo *E. furcellata* transcriptome assembly | | |
| --- | --- | --- |
| Length range | Transcripts number | Unigenes number |
| 300-500bp | 39525 | 25080 |
| 500-1kbp | 35023 | 19869 |
| 1kbp-2kbp | 26134 | 10630 |
| >2kbp | 25584 | 8207 |
| Total number | 126266 | 63786 |
| Total nucleotides（bp） | 170728307 | 68027300 |
| Mean length（bp） | 1352 | 1066 |
| Max length（bp） | 30689 | 30689 |
| N50（bp） | 2245 | 1649 |
| N90（bp） | 529 | 434 |

| **Table S4.** Amino acids for multiple sequence alignments of serpin subfamily and 6 subfamilies of canonical inhibitors of *E.* *furcellata.* | | |
| --- | --- | --- |
| Gene Name | Best blastx match | |
|  | species | Accession number |
| AmSRPN3 | *Apis mellifera* | XP_001122067.2 |
| AmSRPN4 | *Apis mellifera* | XP_003249882.1 |
| AmSRPN5 | *Apis mellifera* | XP_006562425.1 |
| AgSRPN6 | *Anopheles gambiae* | ABJ52806.1 |
| MsSRPN6 | *Manduca sexta* | AAV91026.1 |
| TmSPN93 | *Tenebrio molitor* | BAL03254.1 |
| NvSPI | *Nezara viridula* | CAH1404838.1 |
| ClSPI | *Cimex lectularius* | XP_014260068.2 |
| AtSPI | *Aethina tumida* | XP_049823040.1 |
| AoSPI | *Acanthoscelides obtectus* | CAH1990128.1 |
| HhSPI2 | *Halyomorpha halys* | XP_014273710.1 |
| NvSPI2 | *Nezara viridula* | CAH1399149.1 |
| CsSPI | *Coccinella septempunctata* | XP_044757582.1 |
| MmSPI | *Molorchus minor* | KAJ8980792.1 |
| HaSPI | *Harmonia axyridis* | XP_045468917.1 |
| HhSPI | *Halyomorpha halys* | XP_024216629.1 |
| ClSPI2 | *Cimex lectularius* | XP_014248718.1 |
| CdSPI | *Cloeon dipterum* | CAB3377734.1 |
| BtSPI | *Bemisia tabaci* | XP_018916488.1 |
| IeSPI | *Ischnura elegans* | XP_046394012.1 |
| HhSPI3 | *Halyomorpha halys* | XP_024215529.1 |
| NvSPI3 | *Nezara viridula* | CAH1395342.1 |
| ClSPI3 | *Cimex lectularius* | XP_024082736.1 |
| ZnSPI | *Zootermopsis nevadensis* | XP_021928454.1 |
| CmSPI | *Clunio marinus* | CRK97515.1 |
| FaSPI | *Fopius arisanus* | XP_011305490.1 |
| PrSPI | *Platymeris rhadamanthus* | QHB21529.1 |
| PpSPI | *Pristhesancus plagipennis* | ATU82833.1 |
| LdSPI | *Lethocerus distinctifemur* | ATU82445.1 |

| **Table S5.** Amino acid for construction of phylogenetic tree of thioester-containing proteins (TEP) | | |
| --- | --- | --- |
| Gene Name | Best blastx match | |
|  | species | Accession number |
| AaTep | *Aedes aegypti* | EAT39604.1 |
| DmTep | *Drosophila melanogaster* | NP_523578.1 |
| IsTep | *Ixodes scapularis* | XP_002409560.1 |
| AgTep | *Anopheles gambiae* | AAG00600.1 |
| AmTep | *Apis mellifera* | XP_397416.3 |
| BbC3 | *Branchiostoma belcheri* | BAB47146.1 |
| SpC3 | *Strongylocentrotus purpuratus* | AAC14396.1 |
| GgC3 | *Gallus gallus* | AAA64694.1 |
| MmC3 | *Mus musculus* | AAC42013.1 |
| BmA2M | *Bombyx mori* | XP_012546509.1 |
| AmA2M | *Apis mellifera* | XP_006565503.1 |
| AmA2M1 | *Apis mellifera* | XP_392454.3 |
| TcA2M | *Tribolium castaneum* | EFA07508.1 |
| NvA2M | *Nasonia vitripennis* | XP_001604193.2 |
| OmA2M | *Ornithodoros moubata* | AAN10129.1 |
| IrA2M | *Ixodes ricinus* | ACJ26770.1 |
| HaA2M | *Hasarius adansoni* | BAK64111.1 |
| EbCD109 | *Eptatretus burger* | BAD12264.1 |
| DlCD109 | *Diadumene lineata* | BAJ05272.1 |
| HsCD109 | *Homo sapiens* | NP_598000.2 |
| CiCPAMD8 | *Ciona intestinalis* | XP_009861615.2 |
| BfCPAMD8 | *Branchiostoma floridae* | XP_002586872.1 |
| HsCPAMD8 | *Homo sapiens* | NP_056507.2 |

| **Table S6.** Degree index of serine protease inhibitor protein interaction network in *E. furcellata.* | | | |
| --- | --- | --- | --- |
| Genes | Score | Genes | Score |
| EfSPI37 | 30 | EfSPI32 | 27 |
| EfSPI5 | 29 | EfSPI36 | 27 |
| EfSPI15 | 28 | EfSPI11 | 27 |
| EfSPI3 | 28 | EfSPI19 | 26 |
| EfSPI28 | 28 | EfSPI1 | 26 |
| EfSPI14 | 28 | EfSPI35 | 26 |
| EfSPI9 | 28 | EfSPI21 | 25 |
| EfSPI34 | 28 | EfSPI31 | 22 |
| EfSPI26 | 28 | EfSPI8 | 21 |
| EfSPI29 | 28 | EfSPI2 | 17 |
| EfSPI20 | 28 | EfSPI24 | 9 |
| EfSPI30 | 28 | EfSPI25 | 8 |
| EfSPI13 | 28 | EfSPI16 | 5 |
| EfSPI18 | 28 | EfSPI23 | 5 |
| EfSPI4 | 28 | EfSPI6 | 3 |
| EfSPI17 | 28 | EfSPI38 | 3 |
| EfSPI33 | 28 | EfSPI27 | 2 |
| EfSPI10 | 27 | EfSPI7 | 2 |
| EfSPI22 | 27 |  |  |
